# The Multinational New Ventures on Corporate Performance Under the Work Environment and Innovation Behavior

**DOI:** 10.3389/fpsyg.2022.762331

**Published:** 2022-03-14

**Authors:** Zheng Wang, Ke Zong, Kim Hyun Jin

**Affiliations:** ^1^Xuzhou University of Technology, Xuzhou, China; ^2^School of Economics and Management, TaiShan University, Tai’an, China; ^3^Global Business Administration, Gachon University, Seongnam-si, South Korea

**Keywords:** organizational psychology, corporate environment, innovation, performance, multinational new ventures

## Abstract

To cope with economic globalization and improve the competitiveness of transnational start-ups, the impact of the work environment and innovation behavior on corporate performance of multinational new ventures is analyzed. First, a model of the interaction among environment, innovation behavior, and enterprise performance is proposed. Then, 296 transnational start-ups in coastal areas are surveyed, and the model results are analyzed. Finally, a series of results are obtained. The results show that from the perspective of psychology, work dynamic organizational learning environment has a positive impact on enterprise performance (standardized path coefficient 0.436, *p* < 0.01), and resource environment has a significant positive impact on enterprise performance (standardized path coefficient 0.425, *p* < 0.01). Strategic environment also positively affects enterprise performance (standardized path coefficient 0.474, *p* < 0.01). Therefore, the working environment of multinational new ventures has a positive impact on firm performance, and the mediating function between the working environment and firm performance is firm innovation behavior. With the research to achieve enterprise innovation of multinational new ventures by improving their response to the dynamic environment, the corporate performance has been greatly promoted, and finally, the new ventures would participate in the international market competition.

## Introduction

With the deepening of the degree of global economy, the current economic instability is also increasing year by year, and the uncertain market economic environment is becoming more and more intense. Economic globalization is a double-edged sword. While bringing benefits to China’s development, it also causes problems such as the ideological fluctuation of teenagers and the loss of high-quality talents (Takahashi and Takahashi, 2021). In addition, in the economic development, the first to be affected by the impact is naturally those multinational enterprises in the economic globalization, they may face more serious uncertainty. However, Chinese enterprises are in the period of economic transformation, which is a huge pressure on the survival and development of multinational enterprises (Bass and Grgaard, 2021). For multinational start-ups, the key is improving their comprehensive competitiveness, and the goal is corporate performance. At present, the research on enterprise performance has gone deep into the micro level, and the research is more mature and interdisciplinary with organizational psychology, organizational behavior, social economics, and other disciplines.

The influence of innovation behavior and working environment on multinational enterprises is mainly analyzed, so as to achieve the improvement of corporate performance. First of all, from the perspective of organizational psychology, the working environment of multinational enterprises is deeply analyzed. The relationship among the environment, innovation behavior, and corporate performance is boldly assumed, and then, a verification model is constructed to demonstrate the relationship. Finally, the influence mechanism among the working environment, innovation behavior, and corporate performance of multinational start-ups is obtained. The innovation of this research is to carry out investigation and analysis on multinational enterprises, which is in line with the current development direction of enterprises. Moreover, the construction of verification model can make the argumentation relationship among the influencing factors more accurate. It can help multinational start-ups to improve their performance from the working environment, which will also help to enhance the comprehensive competitiveness of multinational start-ups.

## Literature Review

To deal with economic globalization and enhance the competitiveness of multinational start-ups, many research teams have begun to investigate how to improve the performance of multinational companies. From the perspective of industrial organization psychology and organizational behavior, Ma et al. (2019) reviewed the literature on corporate social responsibility by western micro-scholars in recent years and systematically analyzed the structure, theoretical basis, and results of empirical researches on corporate social responsibility. It solved the structure and suggestions of corporate social responsibility research on the micro level and provided a new research direction for the integration and development of corporate social responsibility theory and micro-enterprise organization theory. Ma and Wu (2020) explored the impact mechanism of improvisational behaviors on new business performance on the basis of resource view and contingency theory. The empirical results using a data sample of 154 new companies showed that the improvisational behaviors of new companies had a positive impact on routines. Routines positively promoted new business performance as well and mediated the relationship between improvisational behaviors and new business performance. Environmental dynamics positively moderated the relationship between improvisational behaviors and new business performance, which was not the mediating role of routines. In the view of dynamic capability theory (Xiong and Wang, 2020) verified the relationship between cultural diversity and the business performance of multinational companies with the global comprehensive investment situation of Chinese listed multinational companies from 2002 to 2016. It was concluded that the internal mechanism of the positive linear relationship between cultural diversity and the performance of multinational enterprises was a dynamic capability mechanism. In addition, if the boundary attribute value of this mechanism was deeply tested, it would be concluded that the longer the established age of multinational innovative enterprises, the deeper their cultural foundation, and the stronger the positive impact of cultural diversity on the performance of multinational start-ups. The richer and more robust the organizational structure of multinational start-ups, the more obvious the positive effect of cultural diversity on the corporate performance. The more single and centralized the ownership structure of multinational enterprises, the weaker the cultural diversity was to promote the corporate performance positively. Lin and Xu (2018) collected relevant information on 246 start-ups from China and found that the factor that promoted innovation was the embeddedness of the organizational structure. Increasing environmental uncertainty would deepen the positive effect of embeddedness on innovation tendency, and the effect of cognitive embeddedness on innovation was promoted in reverse. Akpoviroro et al. (2020) adopted regression analysis, cross-tabulation analysis, and inter-location correlation tests to analyze the cultural and age diversities of the labor force of multinational companies as well as the level of workers’ productivity. It was concluded that the multinational food production industry not only needed ensuring the diversity of their own employees but should also ensure cultural diversity to improve organizational performance.

**Figure 1:**
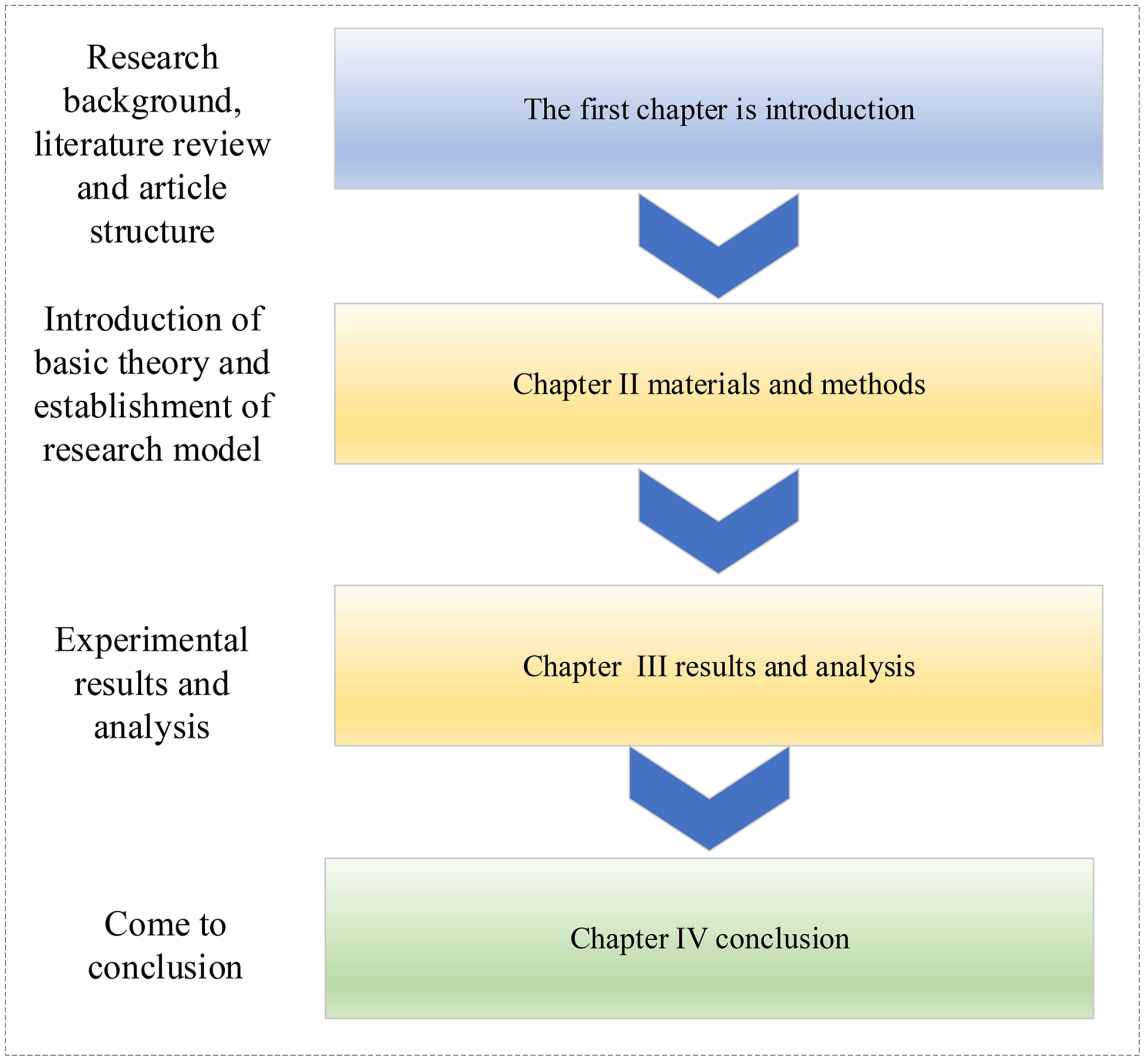
Article organization structure.

From the above literature, it can be found that the various variables between the working environment and corporate performance studied by the researchers are logically broad. Therefore, this focuses on the impact mechanism among working environment, innovation behavior, and corporate performance of multinational start-ups. The specific structure is shown in Figure 1.

Firstly, according to the relevant literature, multinational enterprises, their work environment, innovation behavior, and corporate performance are analyzed from the organizational psychology. Then, the influence model of the interaction among working environment, innovation behavior, and corporate performance of enterprises is established. Questionnaire survey and model analysis are conducted on 296 multinational start-ups in coastal areas, and finally, the relationship among the working environment, innovation behavior, and corporate performance of multinational start-ups is obtained. Thereby, the competitiveness of multinational companies in the international market can be improved.

## Theoretical Analysis and Model Construction of Multinational New Ventures

### Theoretical Analysis of Organizational Psychology

**Figure 2:**
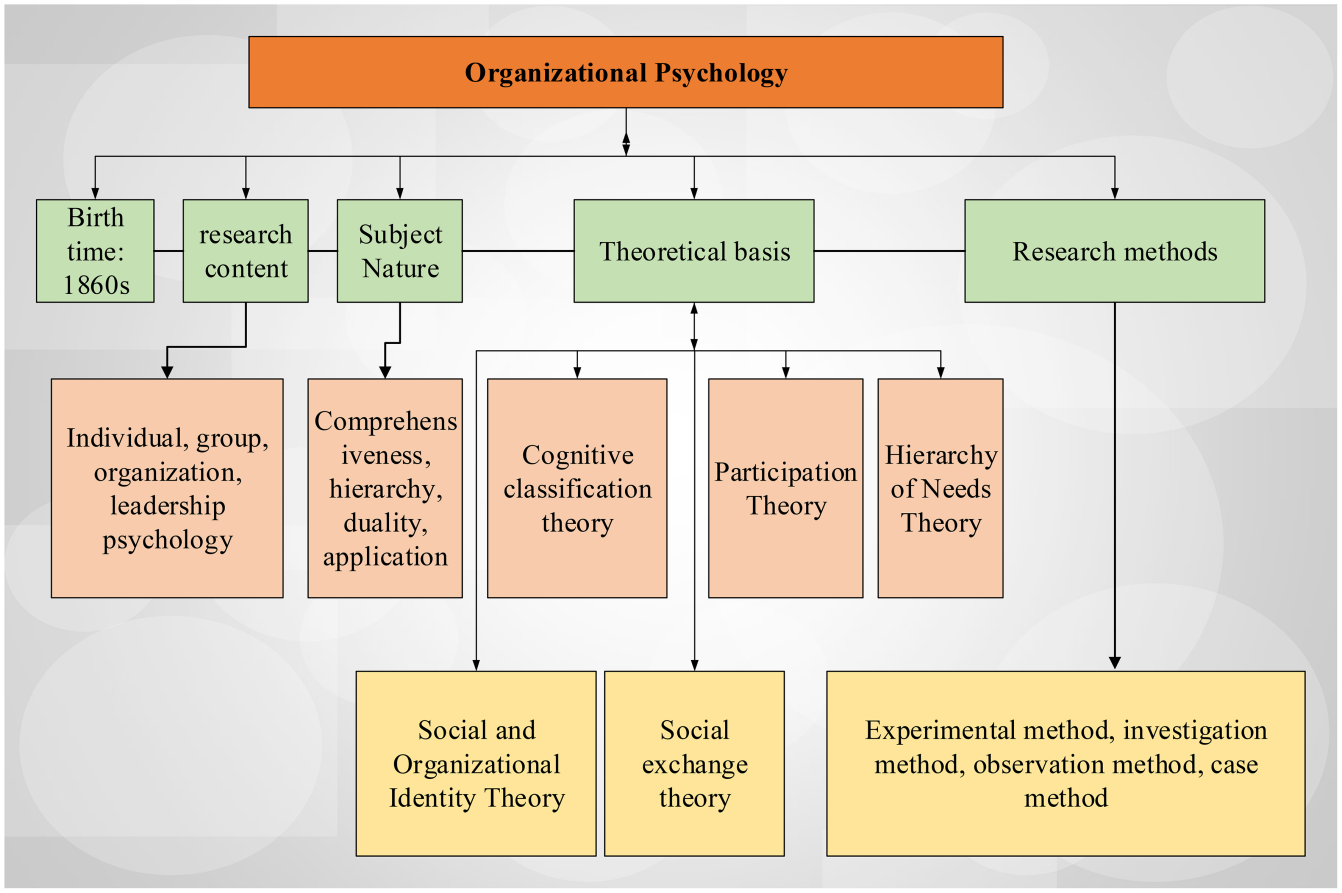
An overview of organizational psychology theory.

Organizational psychology theory contains the following theoretical foundations. Social identity theory: it can innately classify and classify oneself and others, and only in this classification can one’s own consciousness and development develop and change (Qian et al., 2018; Roulin et al., 2021). An overview of the theoretical system of organizational psychology is shown in Figure 2.

Organizational identity theory would be used as an explanatory and reasoning tool. For example, in employee value reasoning related to corporate social responsibility, employees’ views on corporate social responsibility and the awareness of actions are taken to judge whether employees themselves converge with corporate organizational values, which also negatively affects organization members. Cognitive classification theory refers employees are classified and matched according to their own value cognition (Wu and Song, 2019). Social exchange theory combines disciplines like economics, psychology, and sociology, where individuals make different types of activities on the ground of an assessment of risks and benefits under the theoretical fusion. According to the participation theory, the members of the organization are passionately engaged in their own work. In this case, the members of the organization will be more willing to develop toward the combination of their own values, interests, etc., with their work (Rogoza et al., 2018; Chen, 2019). With the fairness theory, people will invariably compare themselves with the members with the same level of the surrounding environment in the organization and psychologically judge the fairness of personal compensation and make judgments that affect work motivation.

### Multinational New Ventures Definition Analysis

The essence of multinational enterprises is carrying out production and operation activities in multiple countries, and their characteristics are that the huge differences in culture and region cause enterprises to face different cultural challenges (Zheng et al., 2018; Wu et al., 2020). Multinational new ventures operate in different cultural environments and need to work hard to learn and discover the hidden benefits of cultural diversity, as well as to understand the relationship between strategy and performance (Gitta et al., 2020; Cao and Wang, 2021). While multinational new ventures carry out business activities, they are accompanied by heterogeneous cultural systems and dynamic environments. The dynamics of this environment come not only from industrial dynamics related to technological change, consumer demand, or competitive activities, but also from the dynamics of the cultural environment caused by international expansion. In general, the capabilities of multinational companies are partly derived from the diverse environment in which they operate and compete. Cultural diversity brings innovative changes to the resource base of multinational new ventures and promotes the formation of dynamic capabilities of multinational new ventures. They can then make effective and market-oriented decisions and systematically solve problems to improve corporate performance.

**Figure 3:**
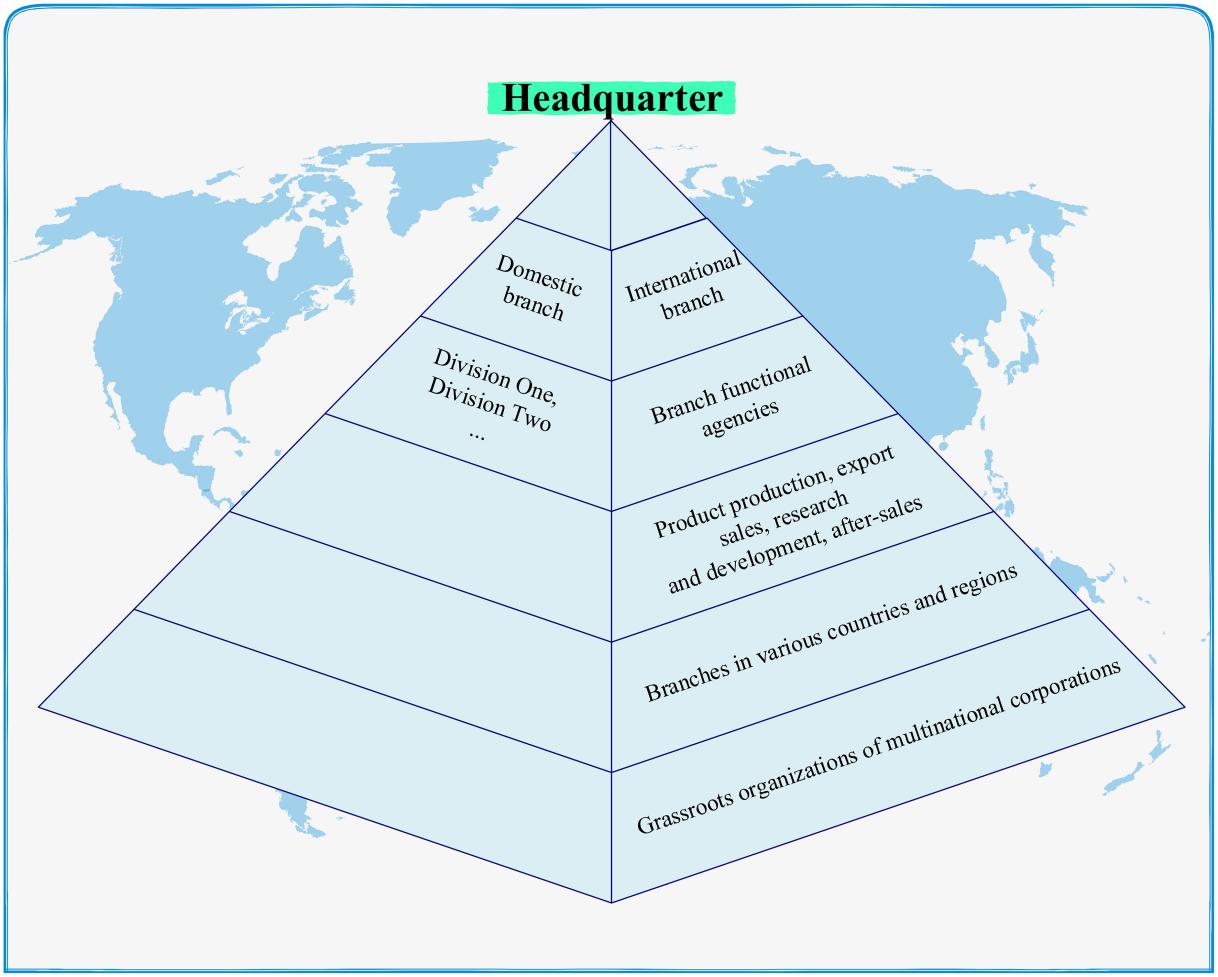
Multinational enterprise gold tower structure chart.

From the perspective of organizational psychology, business organizations will, as always, refer to past experience for development planning during the development process. There are a lot of branches of the theory of multinational enterprise organization life cycle from the perspective of organizational psychology. There is a staged life cycle like an organization, which divides the life cycle of an organization into a growth period in a birth period, an expansion period in a mature period, and a decline period (Wu and Wu, 2017). A similar classification also divides the growth process of a company into four stages, the pre-stage, the entrepreneurial stage, the early development stage, and the growth stage. There are also different definitions for the definition of start-up years. If the start-up is less than 8 years old, or any year between 8 and 12 years old, it can be inferred from the division of years of establishment consciousness that start-ups will inevitably go through a period of running-in before the later stage of stable and mature development. Companies in this period are start-ups. The term multinational new ventures in this article are defined from the perspective of age. The enterprise is in the growth and development stage, and the age of establishment is more than 3 years but less than 10 years. The pyramid organization chart of multinational corporations is shown in Figure 3.

### Work Environment Analysis

Based on the theory of organizational psychology, the working environment of multinational corporations mainly refers to a kind of working atmosphere and working mentality of corporate practitioners in their own organization. Generally speaking, when organizational psychology is adopted to analyze the working environment of the organization members from the micro-behavior psychology, it is easier for the managers of the organization to grasp the psychological process of the organization personnel and the influence mechanism of the organization environment on the personnel in the process of subtle or predictive long-term changes in the organizational environment. Because the corporate “working environment” is closely related to the daily practice of corporate organizations and the process of organizational soundness and standardization, the organizational psychology connotation of corporate “working environment” issues will be more prominent through this model.

From the perspective of organizational psychology, the working environment of an enterprise is specifically the learning environment, resource environment, and strategic environment within the enterprise organization. In addition to the impact of cultural diversity in the working environment of multinational new ventures, one of the main features of the corporate working environment is the dynamic change of the environment. Dynamics will affect a series of behaviors in the business process of the enterprise and ultimately affect the performance of the enterprise. The speed and degree of environmental change, as well as the dynamic uncertainty of enterprise market changes caused by this change in the environment, are summarized as the enterprise dynamic environment (Seo et al., 2021).

### Analysis of Innovation Behavior of Multinational Enterprises

The innovative behavior of multinational new ventures mainly includes the innovation of enterprise business model and the innovative behavior of organization members. The business model is a theoretical framework for the normal operation of how a company or organization makes money and avoids various risks. The value level of business model should include values, value structure, value performance, and value relationship network (Liu and Shen, 2020; Miao et al., 2020). The overall innovation of an enterprise is a summary of the internal innovation of the members of the organization. From the perspective of organizational psychology, the influencing factors of employees’ innovative behavior include the internal and external environment of the organization, members’ own factors, and the cross-influence of individuals and the environment. Factors such as individual employees’ knowledge, potential, intelligence, and way of thinking determine to a large extent their innovative potential and ability. The difficulty of organizing the work of a new venture will affect the innovation needs of the team members. When the work is difficult, employees will spontaneously adjust their existing work models and produce innovative behaviors without completing their personal performance. There will be an inverted U-shaped relationship between work intensity and team members’ innovative behavior. The highest level of innovation is often when the work intensity requires a medium level. At the organizational level, the organization’s innovative behavior will be affected by the organization’s strategy and organizational structure. Group members are more likely to play an extraordinary level of innovation when they are aware of the organization’s innovative incentives.

### Enterprise Performance Content Analysis

**Figure 4:**
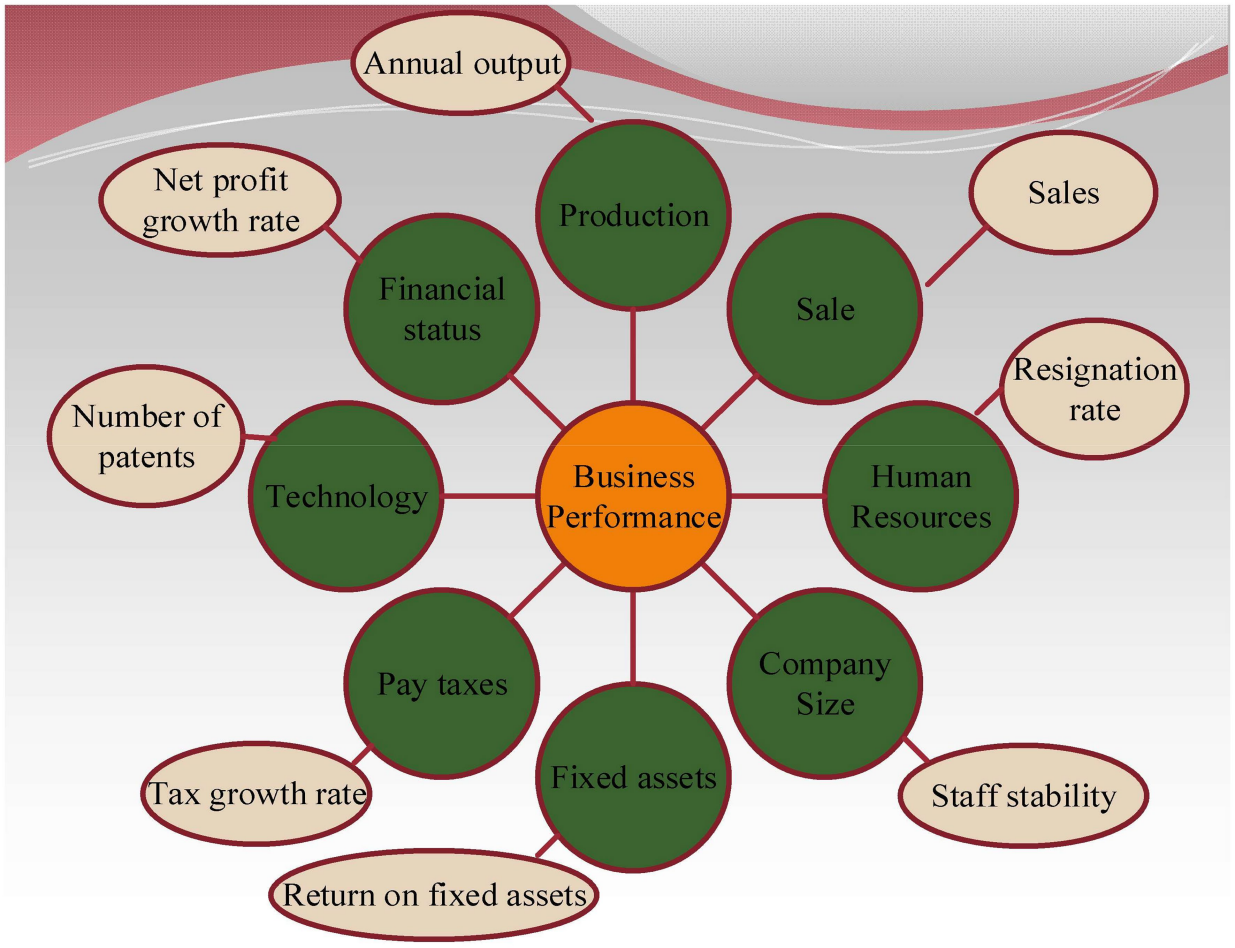
Schematic diagram of corporate performance structure distribution.

From the perspective of organizational psychology, new venture performance refers to the synthesis of value performance and value efficiency created by new venture organizations in the economic activities they engage in by using organizational wisdom. It is usually an important index to measure the completion of corporate strategic goals (Mutonyi and Lien, 2020). The performance indexes of new ventures include sales revenue growth, market share growth, and net profit growth. A schematic diagram of corporate performance distribution is shown in Figure 4.

### Research Methods

(1) Literature method refers to the method of understanding and proving the object to be studied by referring to the literature. The accumulation of literature can save the literature completely and collect the part related to the research topic with emphasis. Data query through CNKI, Google Academic, and other channels and a series of data collection and summary provide a favorable theoretical basis for the research ideas and methods of this work. (2) Comparative analysis refers to the multi-comparison of two or more kinds of research objects, to discover the similarities and differences between them, and to study and learn good methods. This method aims to deeply understand the impact of working environment and innovation behavior on corporate performance of multinational start-ups. (3) Questionnaire survey is a widely used method in social survey. Questionnaire refers to the form used for statistics and surveys to express questions in the form of questions. Questionnaire survey is a method that researchers use this kind of controlled measurement to measure the problems under study, thereby collecting reliable data.

### Hypothesis Presentation

Wiewiora et al. (2020) believed that environmental dynamics emphasize the unpredictability of enterprises in the future caused by the rapid changes in the environment, including customers, market trend rate of change, peer competitors, business growth opportunities, unpredictability in the R&D process, and product innovation. Environmental dynamics affect a series of behaviors in the entrepreneurial activities of new enterprises and thus affect the performance of enterprises (Wiewiora et al., 2020). Liu and Liu (2019) pointed out that the effectiveness of improvisation depends on key moderator variables. Business opportunities are often created by the dynamic external environment. In the face of these opportunities, companies can help promote entrepreneurship through improvisation. Relevant studies also indicated that when the environment is highly dynamic and may cause damage to the enterprise, the occurrence of improvisation is likely to be meaningful (Liu and Liu, 2019). In view of this, the following hypotheses are proposed.

#### Organizational Learning Environment and Innovative Behavior

Organizational learning itself is the process of discovering new opportunities through repetition and experimentation. A good organizational learning environment can cultivate the necessary capabilities of the organization (Nwanekezie et al., 2021). The innovation of multinational new ventures includes continuous change. The company should have static and elastic properties to ensure innovation. The organizational learning environment can become an important driving force for business model innovation. Based on the above analysis, hypothesis is put forward. F1a: the organizational learning environment positively promotes corporate innovation behavior.

#### Resource Environment and Innovative Behavior

In the process of enterprise development, the resource environment in which the enterprise is located is a necessary element of enterprise development and the foundation of the enterprise’s ability to create competitive advantages (Mao, 2019). Only by making use of valuable resources, can an enterprise produce high-efficiency output and provide impetus for its performance. Based on the above analysis, hypothesis is put forward. F1b: resources and environment have a positive impact on innovation.

#### The Relationship Between Strategic Environment and Innovative Behavior

In a competitive business environment, the strategic environment is very important (Serrat, 2019; Cooper et al., 2020). In competition based on business models, competitors can easily copy resources, skills, and even strategies. Advanced strategies can prevent competitors from imitating resources and skills and create obstacles to each other’s strategies. The strategic environment creates a barrier to competition for new strategies adopted to imitate changes in the environment. Based on the above theoretical analysis, the following hypothesis is proposed. F1c: the strategic environment has a positive impact on innovation behavior.

#### The Relationship Between Dynamic Environment and Corporate Performance

The dynamic environment is a generalization of the first three working environments. The dynamic environment systematically uses existing resources while also creating new resources and skills that are closely related to the activities within the organization. The dynamic environment creates new competitive advantages and generates new knowledge, products, and processes that can better contribute to the company’s performance (Yuan and Wu, 2020). After the relevant literatures are summarized and analyzed, the following hypothesis is proposed. F1d: dynamic environment has a positive impact on growth performance.

#### The Relationship Between Corporate Innovation Behavior and Corporate Performance

The business environment is changing rapidly. In an environment of e-commerce turmoil, financial crisis, unstable regulatory conditions, and rapid technological progress, companies must adapt to market conditions and change the way they do business with suppliers, customers, and partners. Innovative adjustment of business model should be done (Staniewski and Awruk, 2019). For a company, innovation means standing out from the competition in the industry and having many new options for planning its own business. Fierce competition and rapid replication of successful business models force all companies to continuously update their business models in order to achieve sustainable competitive advantages. After the relevant literatures are summarized and analyzed, the following hypothesis is proposed. F1e: innovation behavior has a positive impact on performance.

### Model Building

**Figure 5:**
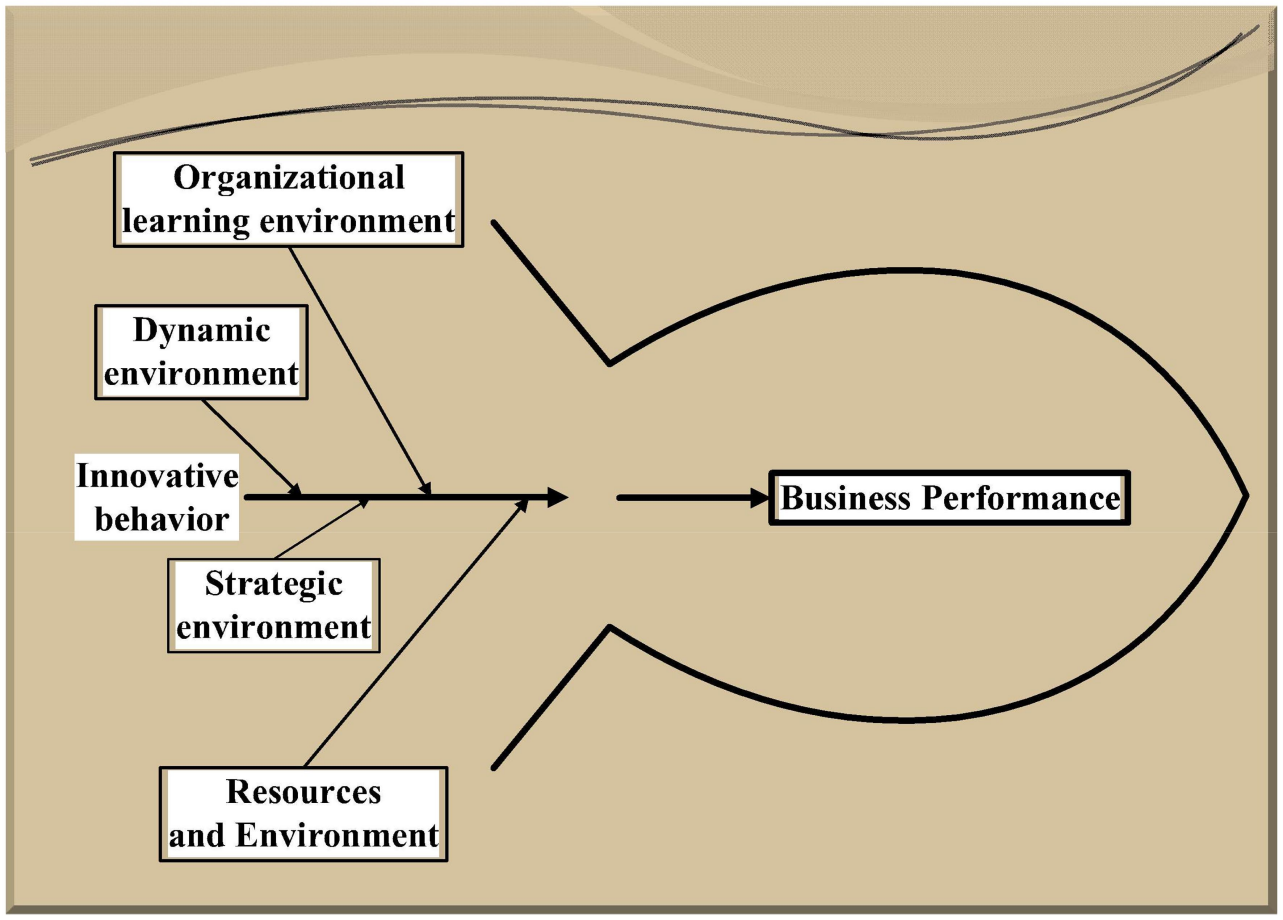
Schematic diagram of model correction.

Based on the above theoretical analysis and research hypotheses, a conceptual model of the research on the impact of the corporate working environment on corporate innovation behavior and corporate innovation behavior on corporate performance is constructed, as illustrated in Figure 5.

From Figure 5, a good organizational learning environment and dynamic resources can promote the innovative behavior of the enterprise, thereby improving the performance of the enterprise. The cutting-edge strategic environment can prevent competitors from stealing corporate resources and skills, and the resource environment can provide motivation for corporate performance. Organizational learning environment, dynamic resources, innovative behavior, strategic environment, and resource environment can all have an impact on corporate performance from different perspectives.

According to the relationship between the variables and the overall model, a structural equation model is selected to verify the fit between the model and the data. Structural equation models allow researchers to map the relationships between hidden variables and determine whether these structural relationships are meaningful. Therefore, this research is suitable for investigating the causal relationship between corporate environment, corporate innovation behavior, and corporate performance. AMOS 20.0 is employed to fit and verify the proposed hypothesis model (Russo et al., 2021).

### Research Design

#### Data Collection

To ensure the reliability of the data, standards are set for the study samples according to the research topic and research question. First, the enterprise must be established for more than 3 years of normal operation and less than 10 years. Second, it must be an independent entity. In this study, questionnaires are mainly used to collect data. Questionnaires are filled out by people familiar with the company, and data are collected through private networks and learning teams. The research samples are mainly from two aspects. One is from a mature authoritative management consulting company, and the other is from the coastal areas of Guangdong and Zhejiang transnational industrial science and technology parks and incubators. Questionnaires for this study are sent out from November 2020 to April 2021. A total of 400 questionnaires are sent out, and 340 are recovered with a recovery rate of 85%. Through the verification of incomplete questionnaires and sampling standards, 296 valid questionnaires are recovered with an effective recovery rate of about 87%, meeting relevant requirements (Zhang et al., 2019).

#### Variable Measurement

In this study, self-report method is mainly adopted to measure variables with 7-point Liken scale (Wu et al., 2019). After purifying of the measurement questions through small sample pre-test, a formal measurement scale is formed, as presented in Table 1.

**Table 1:** Variable factors and measurement items.

**Factors**	**Measurement items**	**Load value**
Organizational learning environment	C11 Enterprise personnel in the organization have a strong sense of self-learning and promotion	0.693 0.855 0.629 0.777 0.682 0.606 0.785 0.682 0.727 0.903 0.530 0.561 0.781 0.709 0.594 0.767 0.901 0.766 0.589 0.676 0.820 0.711 0.745 0.759 0.782 0.696 0.648 0.598 0.365 0.489 0.478 0.965
	C12 The ability and skill level of the personnel in the organization is clear.	
	C13 Strong awareness of learning atmosphere and technology competition within the organization.	
	C14 The organization develops plans and goals for employees’ learning.	
Resources environment	C21 Free communication with foreign countries.	
	C22 Labor resources of multinational new ventures	
	C23 Material resources provided by the parent company	
	C24 Stable production raw materials	
	C25 Political and social stability resources	
	C26 Technical resources support	
	C27 Work resources provided within the organization	
Strategic environment	C31 Match degree with local national policies	
	C32 National life pillar industry	
	C33 Technicians master the core technology.	
	C34 Long-term strategic planning	
Innovation	C41 Innovate products or services for target customer companies	
	C42 Leading the innovation of customer needs	
	C43 Innovation of enterprise core resources	
	C44 Innovation in partner networks	
	C45 Innovation in the way the company earns revenue	
	C46 Innovation of enterprise cost structure	
Business performance	C51 The growth rate of the company’s net profit	
	C52 Growth rate of return on investment	
	C53 Growth in the number of workers	
	C54 New product or new service	
	C55 Core customer growth	
	C56 The growth rate of the company’s market share	

## The Results of the Research on the Relationship Between Multinational New Ventures and Performance Variables

### The Statistical Reliability and Validity Analysis Results of the Questionnaire of the Research Variable Design Model

**Figure 6:**
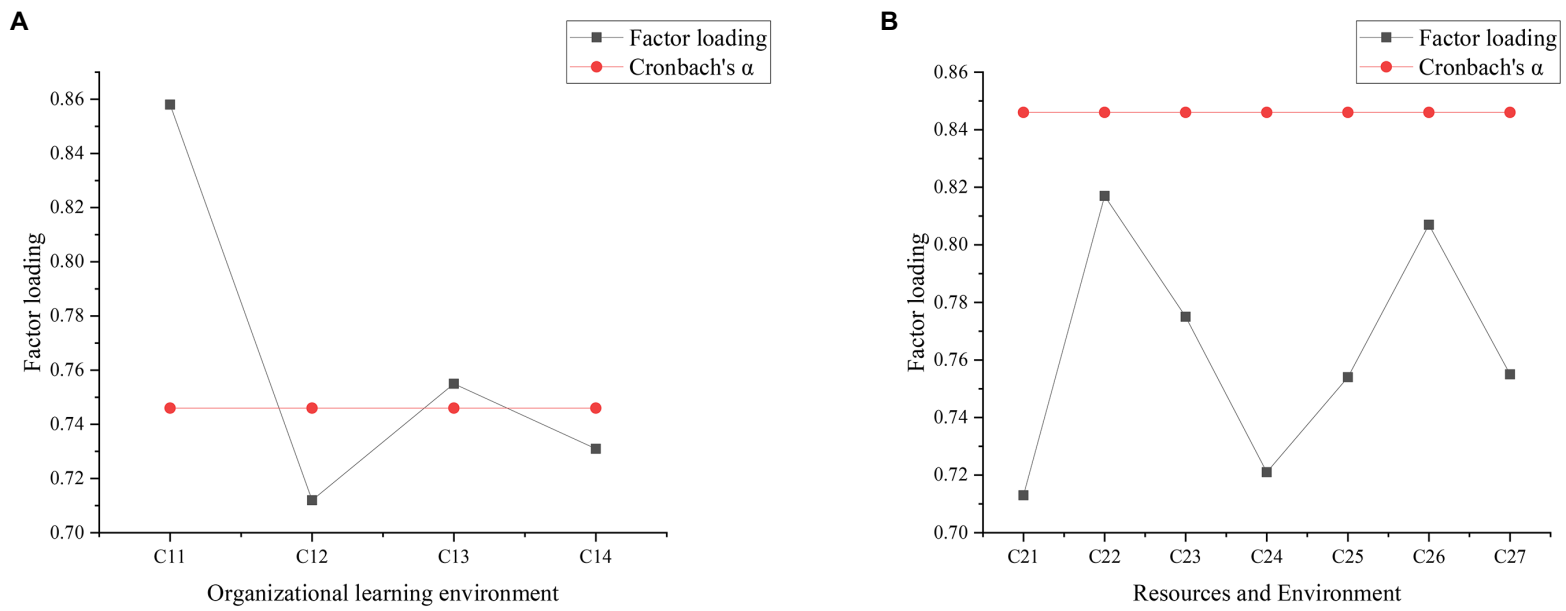
**(A)** Reliability and factor analysis of organizational learning environment variables; **(B)** reliability and factor analysis of resource and environment variables.

**Figure 7:**
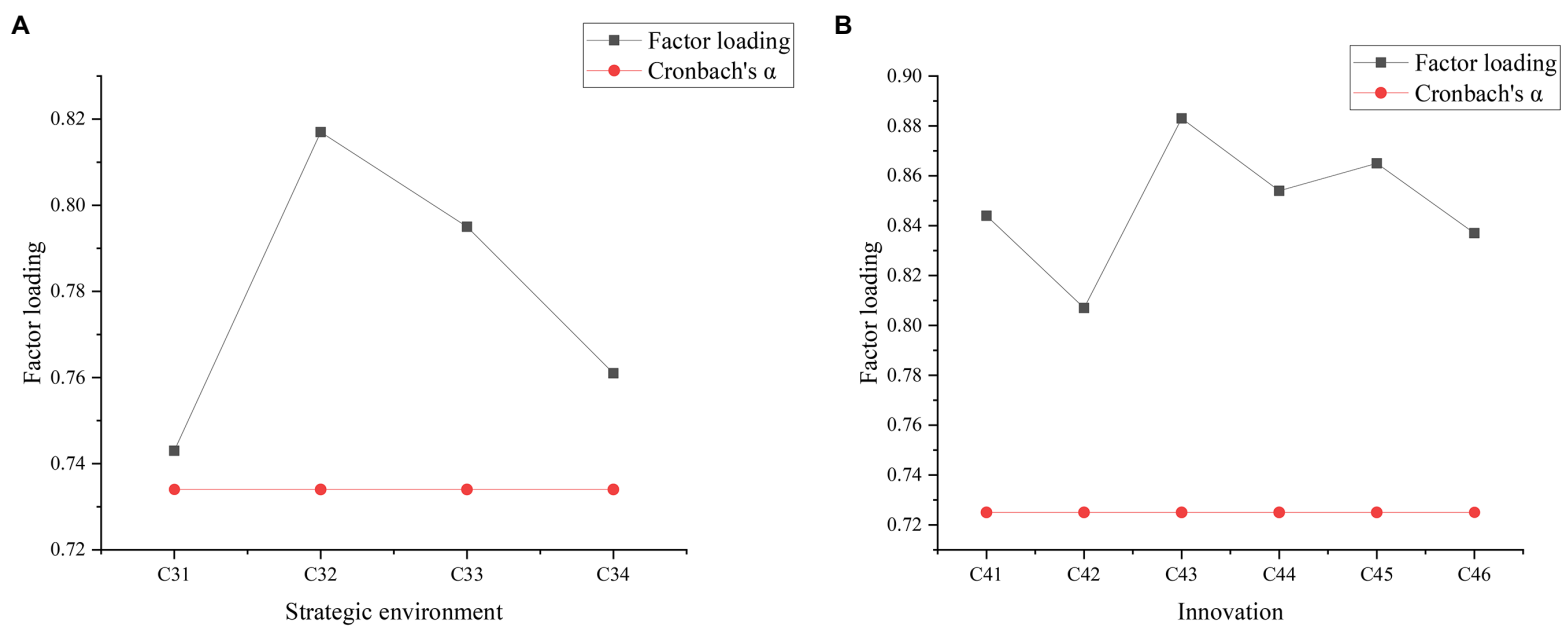
**(A)** Reliability and factor analysis of strategic environment variables; **(B)** reliability and factor analysis of innovation variables.

**Figure 8:**
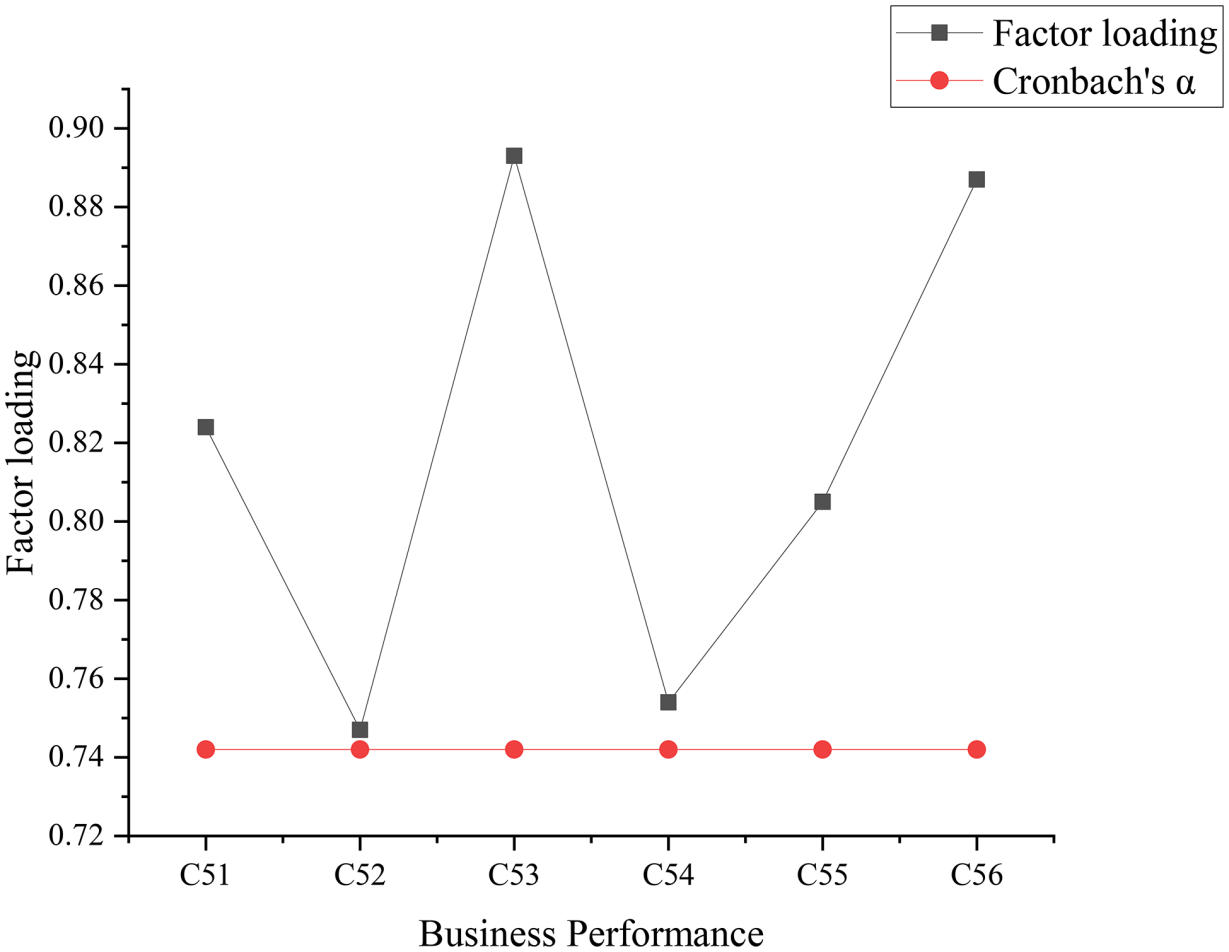
Reliability and factor analysis of enterprise performance variables.

**Figure 9:**
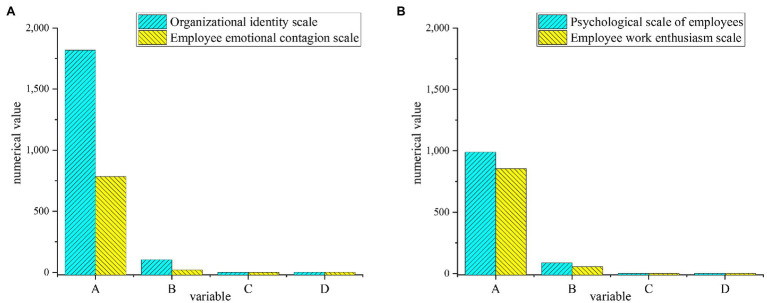
Results statistics of validity analysis. **(A)** Validity analysis of organizational learning environment variables and resource environment variables; **(B)** Validity analysis of strategic environment variables, innovation variables, and enterprise performance variables. (A: approximate chi-square; B: degrees of freedom; C: significance; and D: statistical test of the four scales).

To visually express the reliability analysis and factor loading results of the test factors of each variable (Wu et al., 2018; Yuan and Wu, 2020), the reliability and factor analysis of each variable are shown in Figures 6–8. Figure 9 below represented the validity analysis of the questionnaire.

Figure 6 shows that in the analysis of organizational learning environment and variable reliability and factors, the reliability value of C11 is the largest, and the Cronbach’s α coefficient is about 0.75. In the reliability and factor analysis of resource environment variables, C22 factor has the largest reliability value, and its Cronbach’s α coefficient is about 0.9. In the reliability and factor analysis of the strategic environmental variables shown in Figure 7, C32 has the highest reliability value with the Cronbach’s α coefficient of about 0.735. In the analysis of innovation variables, C43 get the highest reliability value, and its Cronbach’s α coefficient reaches about 0.75. In the analysis of corporate performance variables shown in Figure 8, C53 has the largest reliability value, where the Cronbach’s α coefficient is about 0.75. All the Cronbach’s α coefficient values exceed 0.7, indicating that the variables have better combined reliability. Through the exploratory factor analysis of the sample data and the KMO (sampling suitability) test, the loading value of each factor is above 0.5. The sample data have good convergence and discriminant validity, which indicates that the common variables are not very high, and the causal relationship among work environment, innovation behavior, and corporate performance can be analyzed. The correlation coefficient of each variable is between 0.482 and 0.629, as presented in Table 2.

**Table 2:** Correlation coefficient table of each variable.

	1. Organizational learning environment	2. Resources environment	3. Strategic environments	4. Innovative behaviors	5. Business performance
1	1	0.612	0.523	0.629	0.545
2		1	0.575	0.482	0.605
3			1	0.571	0.594
4				1	0.486
5					1

### Structural Equation Model Analysis Results Among Research Variables

AMOS 20.0 is employed to fit and verify the hypothetical model proposed in this article. The action paths between the variables are numbered with L, with a total of seven paths, as presented in Table 3.

**Table 3:** Statistical table of structural equation model results.

Path	Conclusion (*p* < 0.05)
L1 Innovative behavior←Organizational learning environment	Pass
L2 Innovative behavior←Resources environment	Pass
L3 Innovative behavior←Strategic environments	Pass
L4 Business performance←Innovative behavior	Pass
L5 Business performance←Organizational learning environment	Pass
L6 Business performance←Resources environment	Pass
L7 Business performance←Strategic environments	Pass

**Figure 10:**
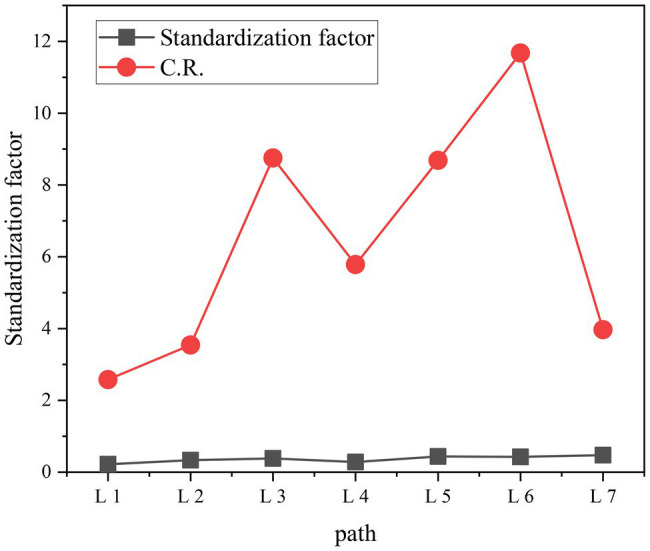
Structural equation model standardization coefficient and CR distribution diagram. CR, construct reliability.

The standardized coefficients and CR values of each path structure equation model are shown in Figure 10.

**Figure 11:**
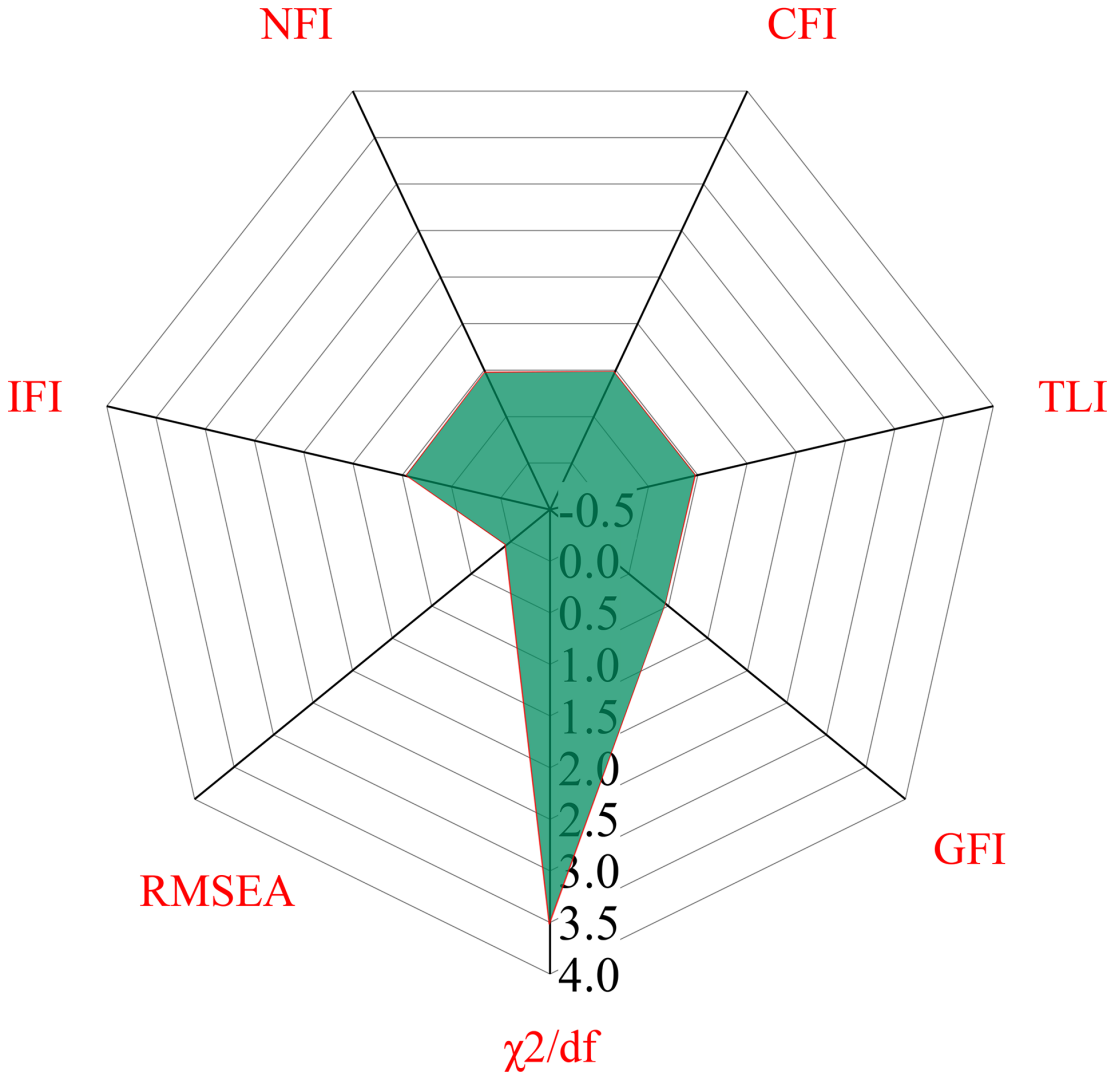
Radar distribution map of model fitting index. GFI, Goodness Fit Index; TLI, Tucker Lewis Index; CFI, Compare Fitting Index; NFI, Normed Fit Index; IFI, Incremental Fit Index; RMSEA, Root Mean Square Error Approximate; *χ*^2^/df, Chi-square/degree of freedom.

The results of the fitting index of each model are shown in Figure 11.

Figure 11 shows that all indexes meet the numerical requirements of model fitting, indicating that the fitting results are good and the model is acceptable after correction.

In summary, the above test results show that the organizational learning environment under the corporate work environment from the perspective of organizational psychology has a significant positive impact on corporate innovation behavior (standardized path coefficient 0.219, *p* < 0.01), so the hypothesis F1a is supported by verification. The resource environment in the corporate work environment has a significant positive impact on innovation behavior (standardized path coefficient is 0.336, *p* < 0.01), so the hypothesis F1b is supported by verification. The strategic environment in the corporate work environment has a significant positive impact on innovation behavior (standardized path coefficient 0.381, *p* < 0.01), so the hypothesis F1c is supported by verification. Innovation behavior has a significant positive impact on corporate performance (standardized path coefficient is 0.284, *p* < 0.01), so the hypothesis F1e is supported by verification. As mentioned above, the dynamic environment of enterprise work is a generalization of the learning environment, resource environment, and strategic environment of the organization. The organizational learning environment has a significant positive impact on corporate performance (standardized path coefficient 0.436, *p* < 0.01). Resources and environment have a significant positive impact on corporate performance (standardized path coefficient 0.425, *p* < 0.01). The strategic environment has a significant positive impact on corporate performance (standardized path coefficient 0.474, *p* < 0.01), so the hypothesis F1d holds.

## Conclusion

It is mainly aimed to investigate the influence of innovation behavior and working environment on the performance of multinational enterprises. From the perspective of organizational psychology, the multinational enterprises as well as their working environment, innovation behavior, and corporate performance, etc., are analyzed. The influence model of the interaction among work environment, innovation behavior, and corporate performance is proposed. A questionnaire survey and model analysis on 296 multinational start-ups in the coastal area are carried out. The results showed that the organizational learning environment, resource environment, and strategic environment in the working environment have a significant positive influence on innovation behavior (standardized path coefficient is 0.219, 0.336, and 0.381, respectively, *p* < 0.01). Besides, innovation behavior, organizational learning environment, resource environment, and strategic environment have the significant positive impact on corporate performance (standardized path coefficient is 0.284, 0.436, 0.425, and 0.474, respectively, *p* < 0.01). However, there are still limitations. The research samples are geographically restricted. Many multinational enterprises in coastal areas are selected, and the samples of multinational enterprises in inland areas are not included. In addition, the empirical research is mainly conducted focusing on the interaction among the dynamic working environment, innovation behavior, and corporate performance of enterprises. No other variables are involved. Aiming at the shortcomings of this research, a prospect for the future is raised. From the perspective of organizational psychology, the interaction among the working environment, innovation behavior, and corporate performance of multinational start-ups is studied. It can expand the sample area of multinational companies and increase the number of years of business establishment as well as factors such as the index of scale of the enterprises.

## Data Availability Statement

The raw data supporting the conclusions of this article will be made available by the authors, without undue reservation.

## Ethics Statement

The studies involving human participants were reviewed and approved by the ethics committee of Gachon University. The patients/participants provided their written informed consent to participate in this study. Written informed consent was obtained from the individual(s) for the publication of any potentially identifiable images or data included in this articles.

## Author Contributions

All authors listed have made a substantial, direct and intellectual contribution to the work, and approved it for publication.

## Conflict of Interest

The authors declare that the research was conducted in the absence of any commercial or financial relationships that could be construed as a potential conflict of interest.

## Publisher’s Note

All claims expressed in this article are solely those of the authors and do not necessarily represent those of their affiliated organizations, or those of the publisher, the editors and the reviewers. Any product that may be evaluated in this article, or claim that may be made by its manufacturer, is not guaranteed or endorsed by the publisher.
